# Anthropometric data as predictors of Obstructive Sleep Apnea severity

**DOI:** 10.1590/S1808-86942011000400017

**Published:** 2015-10-19

**Authors:** José Antonio Pinto, Luciana Ballester de Mello Godoy, Valéria Wanderley Pinto Brandão Marquis, Thiago Branco Sonego, Carolina de Farias Aires Leal, Marina Spadari Ártico

**Affiliations:** 1 Otorhinolaryngologist. Head/Chief; 2 Medical doctor otorhinolaryngologist. Assistant at the Otorhinolaryngology and Head & Neck Surgery Nucleus, São Paulo; 3 Medical doctor. otorhinolaryngologist. Assistant at the Otorhinolaryngology and Head & Neck Surgery Nucleus, São Paulo; 4 Medical resident in otorhinolaryngology - Otorhinolaryngology and Head & Neck Surgery Nucleus, São Paulo; 5 Medical resident in otorhinolaryngology - Otorhinolaryngology and Head & Neck Surgery Nucleus, São Paulo; 6 Medical resident in otorhinolaryngology - Otorhinolaryngology and Head & Neck Surgery Nucleus, São Paulo

**Keywords:** obesity, obstructive, polysomnography, sleep apnea

## Abstract

**Abstract:**

The Obstructive Sleep Apnea Syndrome is a chronic disease characterized by episodes upper airway collapse, and has been associated with increased cardiovascular morbidity.

**Aim:**

To correlate the neck, abdominal and pelvic circumference with the AHI and oxyhemoglobin saturation in OSA patients, and to correlate these values with disease severity.

**Materials and methods:**

A prospective descriptive study of 82 patients evaluated complaints suggesting OSA, from July 2008 to March 2010. All patients underwent polysomnography, an ENT clinical exam, measures of the BMI, abdominal, pelvic, and cervical circumferences. The mean, standard deviations and Spearman's correlations were analyzed.

**Results:**

The mean AHI in men was 39 events/hr; in women it was 21 events/hr in women. The mean neck circumference was 34.5 cm in women and 41.3 cm in men, the mean abdominal circumference was 94.3 cm in women and 101.5 cm in men, and the pelvic circumference was 105.7 cm in men and 108.7 cm in women. The neck circumference correlated more closely to the AHI in men (r=+0.389 *p*=0.001). The relationship between the abdominal circumference correlated more with AHI than with the BMI in men (AbC r=+0.358 *p*=0.003 BMI r=+0.321 *p*=0.009).

**Conclusion:**

The neck circumference is the best anthropometric measurement of respiratory disorder severity compared to the AbC or the BMI.

## INTRODUCTION

The obstructive sleep apnea hyperpnea syndrome (OSAHS) is a chronic disease consisting of partial or complete episodes of upper airway collapse that result in oxygen desaturation and micro-awakenings, causing symptoms ranging from snoring to excessive daytime sleepiness. Aside from these symptoms that negatively affect the quality of life, OSAHS also increases cardiovascular and cerebrovascular morbidity and mortality.

Polysomnography, notwithstanding its limitations, is the gold standard for diagnosing OSAHS. It is a costly procedure that is not readily available - especially in underdeveloped countries - and requires expert technical service. Therefore, a simpler and less costly method is needed as a screening method for OSAHS^1^.

Obesity is considered a predictive factor for OSAHS in many scientific published papers. At present, this condition is classified as visceral (or android), and peripheral (or gynoid); visceral obesity correlates more closely with OSAHS^1^. There are several anthropometric measures for grading obesity, such as the body mass index (BMI), the neck circumference (NC), the abdominal circumference (AC), the hip circumference (HC), and the modified Mallampati index^2^, ^3^, ^4^, ^5^. It is important to define which of these measures is more predictive of obesity and its correlation with OSAHS^2^.

The hypothesis of a correlation between anthropometric measurements and OSAHS was the basis of our study, in which we aimed to investigate this correlation and demonstrate a probable positive predictive factor for OSAHS severity.

## MATERIALS AND METHODS

The study sample consisted of 82 adult patients (66 male and 16 female) that reported disordered sleep (snoring, excessive daytime sleepiness, and possible apnea) in their first medical visit. Patients were seen from July 2008 to March 2010 at our outpatient unit, and were asked to participate in the study. The institutional review board approved the study of 202 patients initially (research protocol 116/010).

We evaluated weight, height, the BMI, NC, AC, HC, and the modified Mallampati index. The NC was measured along a horizontal line across the midline of the thyroid cartilage. The AC and the HC were measured with patients in the standing position. The AC was measured along a point on the upper border of the iliac crest, and the HC was measured along a horizontal line at the femoral greater trochanter. Anthropometric measurements were based on the National Health and Nutrition Examination Survey (NHANES) guidelines.

Full night polysomnography was used in this study. Scalar variables were the apnea hypopnea index (AHI), subdivided into apnea and hypopnea singly, the mean oxygen saturation (SMED), and the minimum oxygen saturation (SMIN), according to the test results.

Patients aged less than 18 years and with an AHI below 5 were not included in the study.

The analysis was made with the SPSS (Statistical Package for Social Sciences) software, version 17.0 - Spearman's correlation analysis, the Mann-Whitney test, and the Kruskal-Wallis test.

## RESULTS

There were 202 patients presenting at our unit with complaints of sleep disorders during the study period. Of these, 120 were excluded because of incomplete data for the study protocol, and an AHI < 5. The resulting sample comprised 82 patients: 66 male (80.5%) and 16 female (19.50%).

Ages ranged from 25 to 71 years (mean = 43.76 years). Table 1 presents the scalar variables in our statistical analysis of the study data, their variations and means. The position of the tongue relative to the palate, evaluated using the modified Mallampati index, revealed 45.1% grade III, 29.3% grade II, 20.7% grade IV, and 4.9% grade I.

**Table 1:** Description of scalar variables.

Variable	n	Minimum	Maximum	Mean	Standard deviation	Percentile 25	Median	Percentile 75
AGE	82	25.00	71.00	43.76	10.62	36.00	43.00	50.00
WEIGHT	82	49.30	126.00	84.61	15.85	73.00	84.00	95.25
HEIGHT	82	1.49	1.93	1.72	0.09	1.65	1.72	1.80
BMI	82	19.26	43.41	28.42	4.56	25.50	28.08	30.94
NC	82	30.00	50.00	39.87	4.10	37.00	40.00	42.50
AC	82	84.00	142.00	100.06	17.45	91.25	101.00	108.50
HC	82	121.00	135.00	106.14	15.02	100.00	107.00	113.00
AHI	82	5.10	108.90	36.08	28.84	12.20	24.40	52.75
HIPO	82	0.30	71.00	18.50	15.36	7.65	13.10	22.55
AP	82	0.00	87.40	17.56	22.35	2.25	6.55	23.79
S MED	82	87.00	98.00	92.81	2.25	92.00	93.00	94.30
SMIN	77	53.00	94.00	80.44	8.58	75.50	82.00	86.00

ALT= height; BMI= body mass index; NC= neck circumference; AC= abdominal circumference; HC= hip circumference; AHI= apnea hypopnea index; HIPO= hypopnea index; AP= apnea index; SMED= mean oxygen saturation; SMIN= minimum oxygen saturation.

Table 2 shows the application of Spearman's correlation analysis to check the relationship between the variables age and anthropometric measurements and the variables associated with OSAHS severity grades.

**Table 2:** Correlation of anthropometric measurements with OSAHS severity.

Variable	Statistics	AHI	SMED	SMIN
	Correlation Coefficient (r)	+0.054	+0.016	-0.122
AGE	Significance (*p*)	0.627	0.885	0.289
	N	82	82	77
	Correlation Coefficient (r)	+0.342	-0.204	-0.244
WEIGHT	Significance (*p*)	0.002	0.066	0.032
	N	82	82	77
	Correlation Coefficient (r)	v0.156	-0.027	-0.087
HEIGHT	Significance (*p*)	0.162	0.809	0.454
	N	82	82	77
	Correlation Coefficient (r)	+0.306	-0.259	-0.239
BMI	Significance (*p*)	0.005	0.019	0.036
	N	82	82	77
	Correlation Coefficient (r)	+0.411	-0.137	-0.283
NC	Significance (*p*)	< 0.001	0.219	0.013
	N	82	82	77
	Correlation Coefficient (r)	+0.393	-0.287	-0.276
AC	Significance (*p*)	< 0.001	0.009	0.015
	N	82	82	77
	Correlation Coefficient (r)	+0.236	-0.258	-0.141
HC	Significance (*p*)	0.033	0.019	0.222
	N	82	82	77

ALT= height; BMI= body mass index; NC= neck circumference; AC= abdominal circumference; HC= hip circumference; AHI= apnea hypopnea index; SMED= mean oxygen saturation; SMIN= minimum oxygen saturation.

A statistically significant correlation was found among the variables weight, BMI, NC, AC, and HC relative to the polysomnography parameters. Thus, we found that the severity grade of the AHI correlated with increased weight and BMI, and with longer NC, AC, and HC. The most significant relations were between the AHI and the scalar variables NC (r+0.411) and AC (r+0.393). The most significant of these, based on the correlation coefficient, was the NC (r+0.411). The SMED correlated positively with the BMI, AC, and HC, of which the most significant relation was with the AC. The SMIN related significantly with weight, BMI, NC, and AC.

The Mann-Whitney test showed that male patients had higher AHIs (*p*<0.05) than females in our search for gender differences in the variables AHI, SMED, and SMIN. However, there were no gender differences in oxygen saturation, as shown on Table 3.

**Table 3:** Comparison among sex and polysomnography parameters.

Variable	Sex	n	Mean	Standard deviation	Minimum	Maximum	Percentile 25	Median	Percentile 75	Sig. (*p*)
	F	16	23.75	21.06	5.40	76.10	9.40	16.90	28.85	
AHI	M	66	39.07	29.79	5.10	108.90	13.62	28.50	54.95	0.042
	Total	82	36.08	28.84	5.10	108.90	12.20	24.40	52.75	
	F	16	92.87	2.56	89.00	98.00	91.00	92.50	95.00	
S MED	M	66	92.79	2.19	87.00	98.00	92.00	93.00	94.00	0.995
	Total	82	92.81	2.25	87.00	98.00	92.00	93.00	94.30	
	F	16	80.44	8.76	65.00	94.00	74.25	80.00	88.50	
S MIN	M	61	80.44	8.60	53.00	92.00	77.00	83.00	86.00	0.697
	Total	77	80.44	8.58	53.00	94.00	75.50	82.00	86.00	

AHI= apnea hypopnea index; SMED= mean oxygen saturation; SMIN= minimum oxygen saturation.

The Kruskal-Wallis test revealed a positive correlation among the AHI, SMED, SMIN, and the Mallampati grade (Table 4).

**Table 4:** Correlations among the modified Mallampati index and polysomnography parameters.

Variable	MLPT	n	Mean	Standard deviation	Minimum	Maximum	Percentile 25	Median	Percentile 75	Sig. (*p*)
	I	4	7.15	0.49	6.60	7.70	6.68	7.15	7.63	
	II	24	39.42	32.14	5.40	94.60	12.20	24.70	53.00	
AHI	III	37	38.38	30.36	5.10	108.90	15.22	28.50	60.20	0.024
	IV	17	33.19	20.13	8.57	76.10	15.90	24.40	49.40	
	Total	82	36.08	28.84	5.10	108.90	12.20	24.40	52.75	
	I	4	94.25	0.96	93.00	95.00	93.25	94.50	95.00	
	II	24	91.75	2.27	87.00	95.00	90.00	92.00	93.00	0.007
SMED	III	37	93.56	1.79	88.30	98.00	92.30	94.00	94.75	
	IV	17	92.33	2.65	89.00	98.00	89.95	92.00	94.30	
	Total	82	92.81	2.25	87.00	98.00	92.00	93.00	94.30	
	I	4	88.50	3.11	85.00	92.00	85.50	88.50	91.50	
	II	23	78.35	9.10	59.00	90.00	70.00	82.00	85.00	
SMIN	III	33	82.61	7.42	53.00	93.00	81.00	83.00	87.50	0.007
	IV	17	77.18	8.95	57.00	94.00	72.00	78.00	83.00	
	Total	77	80.44	8.58	53.00	94.00	75.50	82.00	86.00	

MLPT= modified Mallampati index; AHI= apnea hypopnea index; SMED= mean oxygen saturation; SMIN= minimum oxygen saturation.

## DISCUSSION

An anthropometric study is used to assess obesity by measuring physical variations and global bodily composition. It is recommended as the best parameter for evaluating the nutritional status of population groups; both individuals and groups can be assessed. (WHO, 2004).

The most common parameters in anthropometric studies comprise primary measurements (used singly) such as weight, height, skin folds, and circumferences, and secondary measurements (combined) such as the BMI, the ideal weight, the sum of skin folds, and others.

We used circumference measurements - the NC, HC, and AC - and the combined BMI measurement, relating each with OSAHS severity. As in other published studies, obesity measurements correlated significantly with sleep disorders. Davies & Stradling (1990) demonstrated that the relation between obesity and OSAHS may be explained by variations in the NC, which results from fat deposition in the neck.^6^ In 1991, these authors confirmed that the NC is the best clinical predictor of OSAHS compared to other anthropometric measurements^3^. Deegan & McNicholas (1996) concluded that the AC correlated best with sleep disorders^5^.

As in older papers, Davidson & Patel recently (2008) found that the relation between the NC and AC and the AHI was significant; among these, the AC was more significant (*p<*0.001)^2^. Dixon et al. (2003) considered the NC as a better predictive measurement for the AHI^7^.

We found a significant relation among the variables NC, AC, and HC, and OSAHS severity. The most significant of them in our data was the NC (*p*<0.001), followed by the AC (*p*<0.001). These findings confirm other published results, suggesting that circumference measures are predictive factors for OSAHS severity.

Friedman analyzed anthropometric parameters in relation to postoperative improvement of the AHI in OSAHS patients, and found that surgery was more successful in lean compared to obese patients. Furthermore, his review of the clinical histories of patients revealed that obese subjects tended to develop sleep disorders^8^.

The BMI, which is related with morbidity and mortality but does not reveal the bodily composition, is easy to measure. Because there is much data on body mass and height, it is used as an indicator of nutrition status in epidemiological studies either singly or with other measurements - until measurements of bodily composition are developed for epidemiological surveys^9^.

In a study using the BMI and circumference measurements, Hoffstein & Mateika found that apneic patients had significantly higher BMIs and NCs compared to non-apneic patients. These authors suggested that the BMI is a determining factor for AHI^4^. Ogretmenoglu et al. have suggested that the BMI together with body fat correlate more closely with the AHI. Contrasting with most published papers, these authors believe that the NC correlates poorly with the OSAHS^1^. Katz et al. found that the BMI, age, the internal laryngeal circumference, and NC were significant predictors of OSAHS^10^; they argued that the NC is the most important measurement as a parameter for OSAHS.

We confirmed these studies in that the BMI - with a mean value of 28.42 - was significantly related with the AHI (*p*<0.005), again demonstrating the importance of anthropometric measurements as predictive parameters for OSAHS severity. We also showed significant relations between the BMI, NC, and AC, and the SMIN. The SMED correlated positively with the AC, BMI, and HC, of which the most significant correlation was with the AC (*p<*0.009).

Several published papers have correlated obesity with cardiovascular disease, the metabolic syndrome, and insulin-resistant type II diabetes. Curiously, sleep disorders are not included as obesity-related conditions, although this relationship is well known to experts in sleep medicine. Part of the reason for this is that the mechanism by which fat causes sleep disorders is unclear^2^.

Davidson & Patel have suggested that the Mallampati index evaluates fat deposition in the tongue, and have used this variable as a measure of obesity. These authors found that the Mallampati index correlated with the AHI in males^2^.

In a study of cadavers, Nashi et al. found that the tongue was more voluminous in patients with abdominal obesity. These authors showed that the tongue is a fat deposition site in the oropharynx, and that the percentage of fat in the posterior third of the tongue is more pronounced than in its anterior two thirds. They also found that an increased tongue volume is related with a higher Mallampati index, which in turn correlated with a high prevalence and severity of sleep disorders^11^. We also used the Mallampati index as an anthropometric measurement in our series, and found a significant relationship between this index and polysomnography measurements such as the AHI, SMED, and SMIN.

Bioimpedance is another scientifically validated method for diagnosing obesity; it consist of passing low intensity electrical conduction (800 microA, 50 kHz) across the body to measure the resistance of tissues, total body water, the percentage of fat, and the lean body mass. Several papers have demonstrated that this parameter is superior to the BMI to measure body fat.^1^

## CONCLUSION

The association between obesity and OSAHS is well-known. Controversies arise regarding the most significant predictive parameters. Our study of 82 patients showed that the AHI correlates with increased BMI, NC, AC, HC, and the modified Mallampati index. Of these, the most significant parameter for estimating OSAHS severity was the neck circumference (NC).
